# Supplementary material for: Harbinger transposon insertion in ethylene signaling gene leads to emergence of new sexual forms in cucurbits
Source: Nat Commun. 2024 Jun 7;15:4877. doi: 10.1038/s41467-024-49250-9 (PMC11161486; doi:10.1038/s41467-024-49250-9)
Supplement: Supplementary file 1 — Supplementary Information [file 41467_2024_49250_MOESM1_ESM.pdf]

a

	Nb. Plant	Andromonoecious	Androecious	chi-square	p-value
CAM_WT	10	10	0		
CAM106	10	0	10		
CAM106 x CAM_Sister F2 progeny	200	147	53	0.088889	0.7656

b

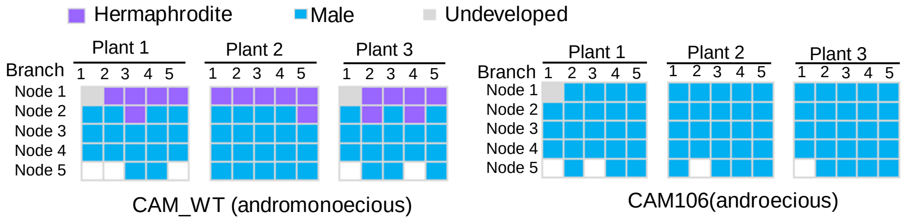

c

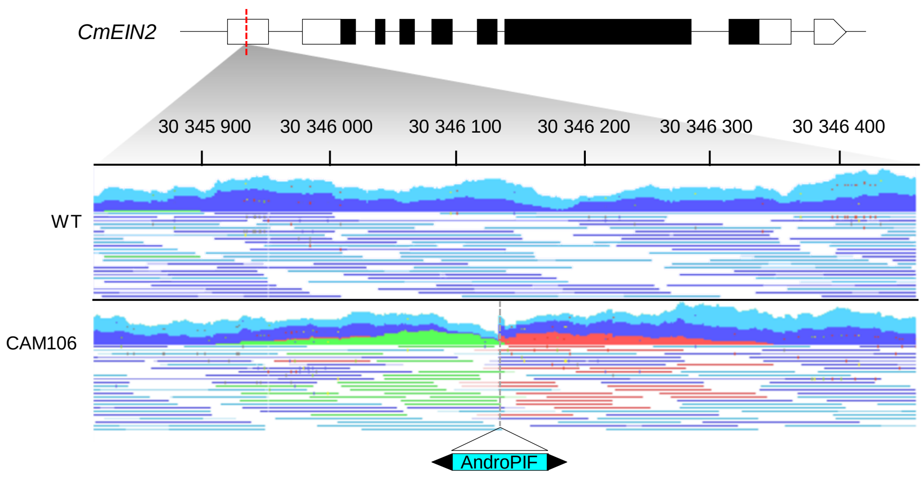

d

Sexual morphs	Nb. Plant	Plants homozygous for *AndroPIF* insertion
CAM_WT (Andromonoecious)	10	0%
CAM106 (Androecious)	10	100%
Andromonoecious F2 plants	150	0%
Androecious F2 plants	57	100%

Figure S1. Sexual morphs observed in androecious *CAM106* spontaneous mutant and andromonoecious CAM_WT line.
a Segregation analysis of sexual morphs in CAM_WT x CAM106 F2 progeny. Nb, total number of plants. **b** Graphic presentation of flower sexual types observed in CAM_WT and in CAM106 lines. **c** Screenshot of CLC Genomics Workbench software showing the insertion of *AndroPIF* into 5' UTR of *CmEIN2*. Discordant reads are shown in green and red color while paired reads are shown in blue and purple. d, PCR screening for *AndroPIF* insertion in F2 plants, described in a, and plants harboring recombination linked to *a3*.

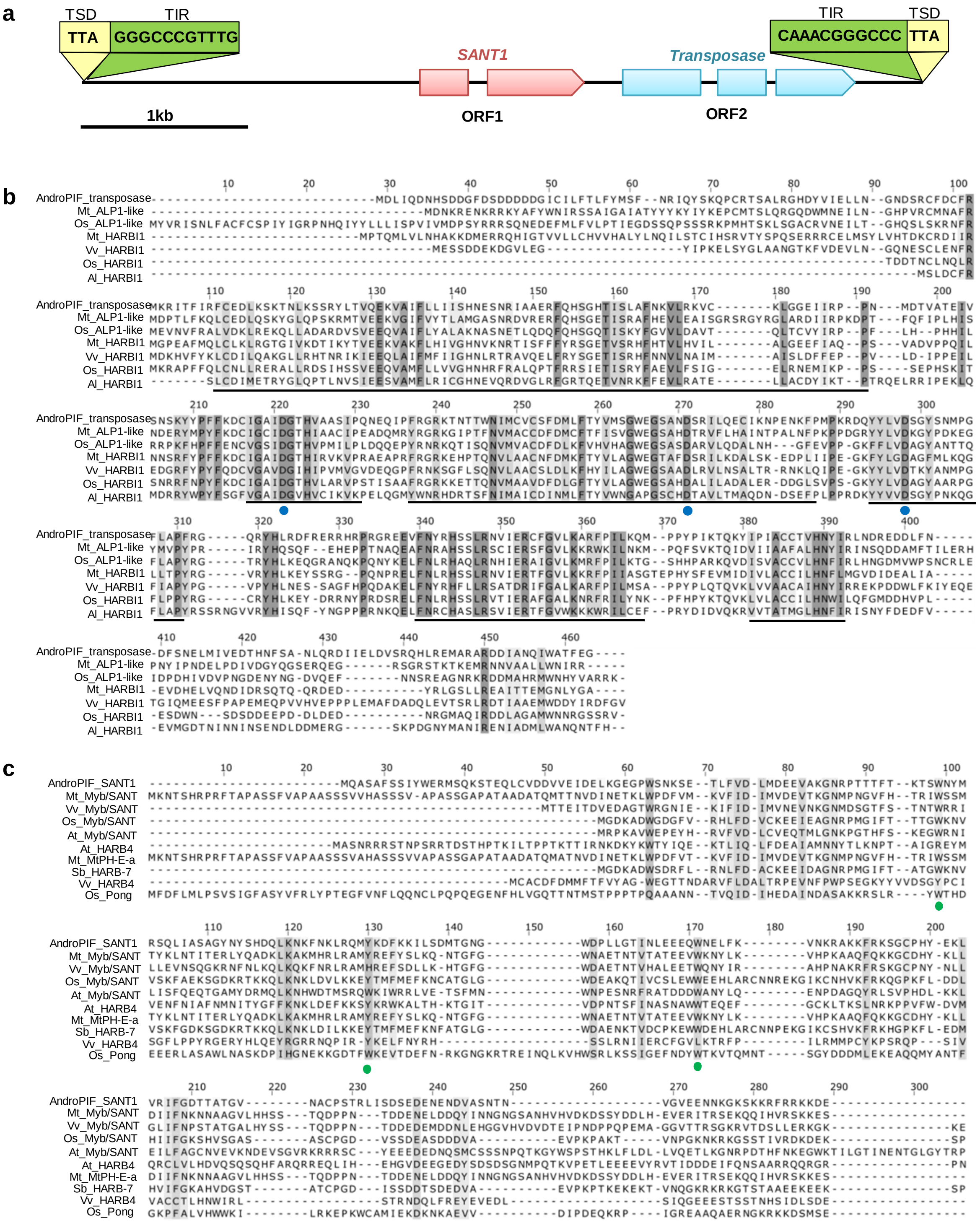

Figure S2. *AndroPIF* is a class II DNA transposon encoding 2 ORFs, SANT1 and Transposase.
a Structural representation and annotation of the *AndroPIF* transposon inserted at the *CmEIN2* locus. Picture is scaled. The 3 bp Target Site Duplication (TSD), and the Terminal Inverted Repeats(TIR) are shown. **b** Amino-acids sequence comparison of *AndroPIF* transposase, and ALP1 and HARBI1 proteins of *M. truncatula* (Mt), *O. sativa* (Os),*V. vinifera* (Vv), and *A. lyrata* (Al). The conserved regions are underlined and the conserved DDD residuals are highlighted by blue dots. **c** Amino-acids sequence comparison of *AndroPIF* SANT1/Myb protein, and homologous proteins from *M. truncatula, V. vinifera, S. bicolor (Sb),* and *O. sativa*. The conserved W residuals are highlighted by green dots.

a

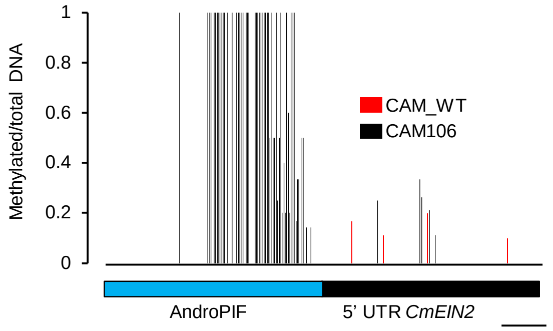

b

	Sequence context	Cytosine number	% of methylation	
			CAM_WT	CAM106
500 bps before	CG	10	0.00%	0.00%
	CHG	19	0.00%	0.76%
	CHH	131	0.15%	0.34%
500 bps in	CG	10	0.00%	28.57%
	CHG	32	0.00%	65.91%
	CHH	99	0.00%	40.91%
500 bps after	CG	12	1.11%	10.53%
	CHG	26	0.53%	0.43%
	CHH	127	0.21%	0.16%

Figure S3. DNA methylation analysis of *AndroPIF* and *CmEIN2* 5'UTR in CAM_WT and CAM106 mutant
a Bisulphite sequencing of 1kb region overlapping *AndroPIF* and *CmEIN2* 5'UTR. The percentage of methylated cytosine is indicated by vertical bars. **b** Percentage of methylated cytosine in CGN, CHG and CHH sequence context for the CAM_WT and CAM106 . Data on 500bps before, within and after *AndroPIF* insertion site is shown.

a

Genotype	Mutation location	Type of transcript	Percentage of transcript	Predicted protein length (aa)
WT	-	-	100%	1291
ein2-SP1	G1175A	Intron 3&4 retention	30%	133
		Intron 3 retention	47%	172
		Exon 4 skipping	23%	1220
ein2-SP2	G1246A	Intron 3 retention	27%	172
		Cryptic acceptor site	73%	172

b

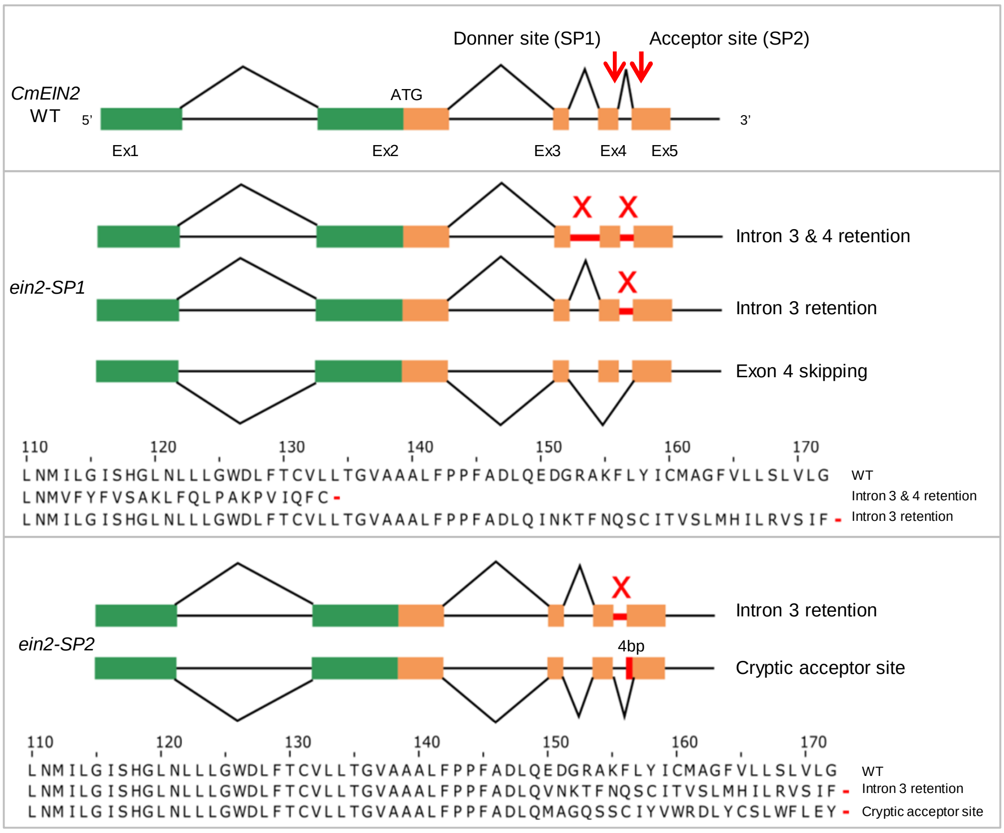

Figure S4. Transcript forms of *CmEIN2* splicing mutant detected by reverse transcription PCR analysis
a Splicing forms of *ein2-SP1* and *ein2-SP2* transcripts in percent. Transcript variations were detected by reverse transcription PCR analysis of leaf mRNAs. The splicing forms were validated by Sanger sequencing, and quantification was carried out using imageJ analysis. **b** Schematic representations of the splicing forms, and predicted protein sequences. Red arrow indicated the mutations located in donner (*ein2-SP1*) and acceptor site (*ein2-SP2*).

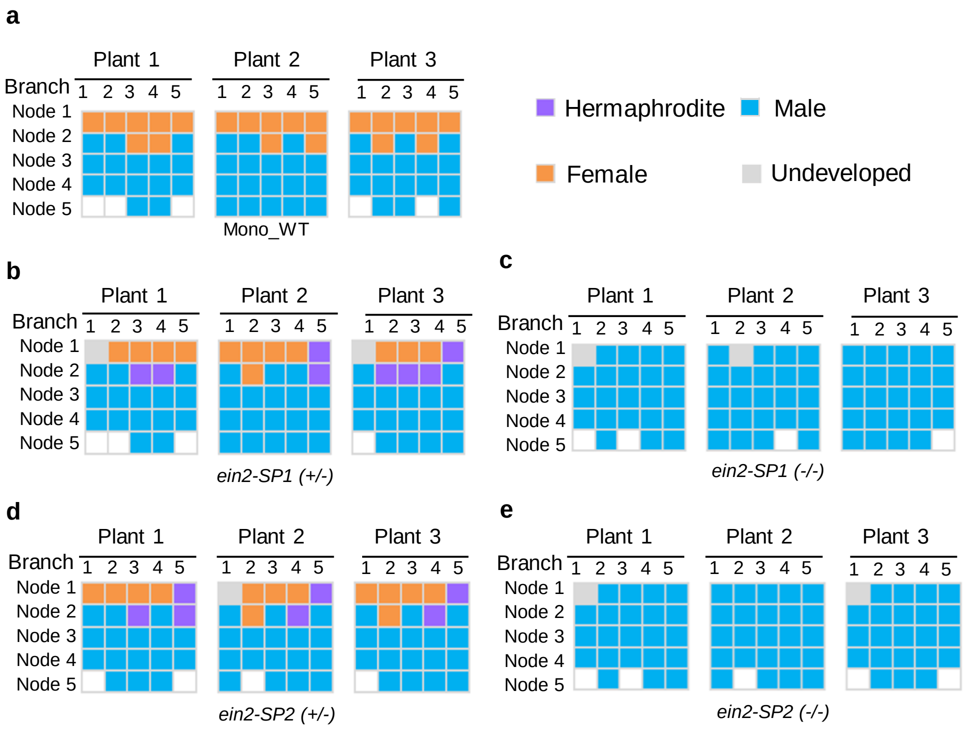

Figure S5. Sexual morphs observed in *CmEIN2* TILLING mutants.
Graphic presentation of flower sexual types observed in wildtype monoecious melon (**a**), heterozygous *ein2-SP1 (+/-)* mutant (**b**), homozygous *ein2-SP1 (-/-)* mutant (**c**), heterozygous *ein2-SP2 (+/-)* mutant (**d**), and homozygous *ein2-SP2 (-/-)* (**e**) mutant.

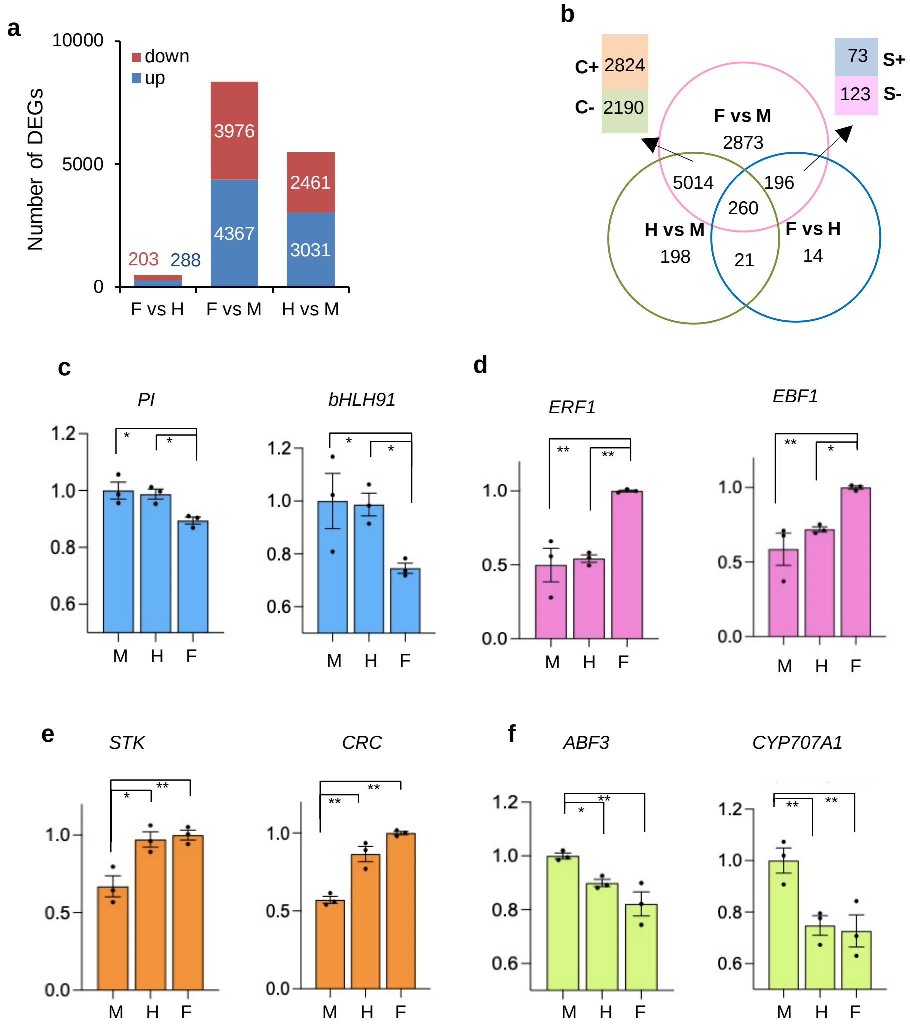

Figure S6 Transcriptome analysis of female, hermaphrodite and males flowers
a Number of differentially expressed genes (DEGs) in pairwise comparison groups. F, female (*wip1*), H, hermaphrodite (*wip/ ein2* double mutant), M, males (acs7/ *ein2* double mutant) flowers. **b** Venn diagram showing overlap between the pairwise comparison groups. **c-f** qPCR analysis of selected candidate genes associated with stamen development (**c**) , S+; stamen inhibition (**d**), S-; carpel development (**e**), C+; and carpel inhibition (**f**), C-. Data are mean ± SE, *P < 0.05, **P < 0.01, two-way ANOVA (with Tukey's post hoc test). M, Male flowers; H, hermaphrodite flowers; F, Female flowers. n= 3 biological replicates.

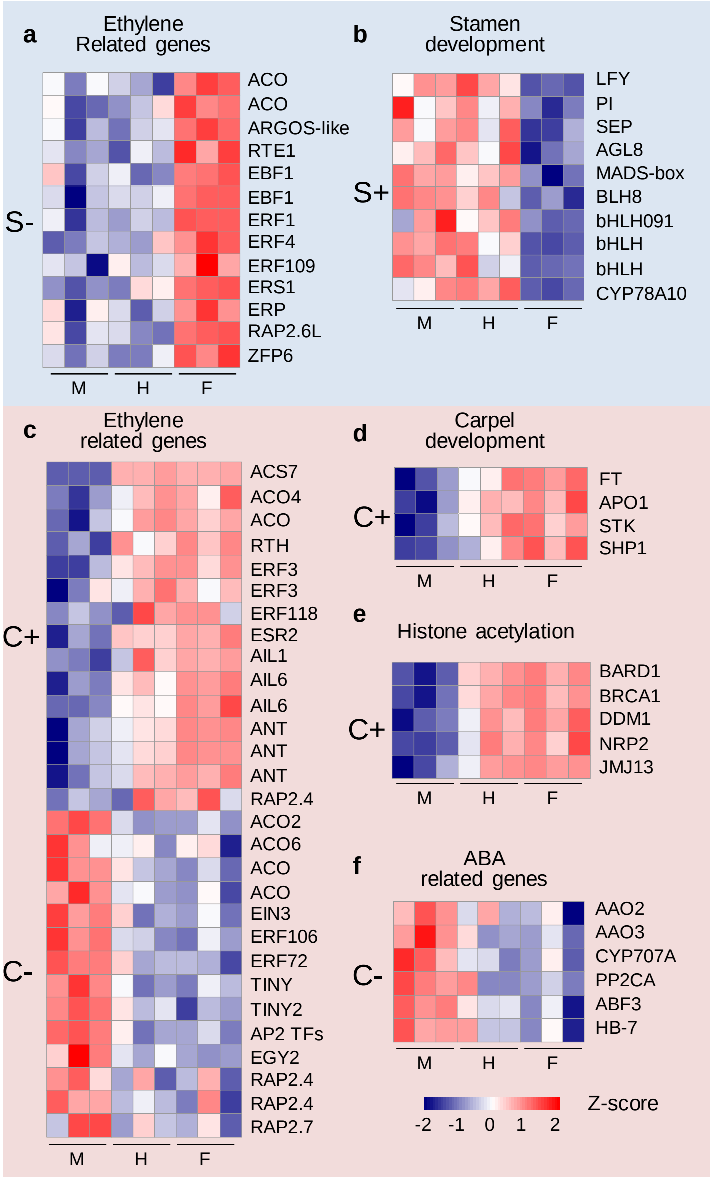

Figure S7. Differential gene expression analysis between female (*wip1*) , hermaphrodite (*wip1/ein2*) and male (*acs7/ein2*) flowers. **a** Heat maps of E- ethylene-related genes differentially expressed in female compared to hermaphrodite and male flowers. The *z*-score scale represents mean-subtracted regularized log-transformed read counts. **b** Heat maps of E+ genes differentially expressed in female compared to hermaphrodite and male flowers. **c** Heat maps of C+ (up) and C- (down) ethylene-related DEGs expressed in male compared to hermaphrodite and female flowers. **d** Heat maps of C+ DEGs expressed in male compared to hermaphrodite and female flowers. **e** Heat maps of C+ brassinosteroid related DEGs expressed in male compared to hermaphrodite and female flowers. **f** Heat maps of C- ABA related DEGs expressed in male compared to hermaphrodite and female flowers.

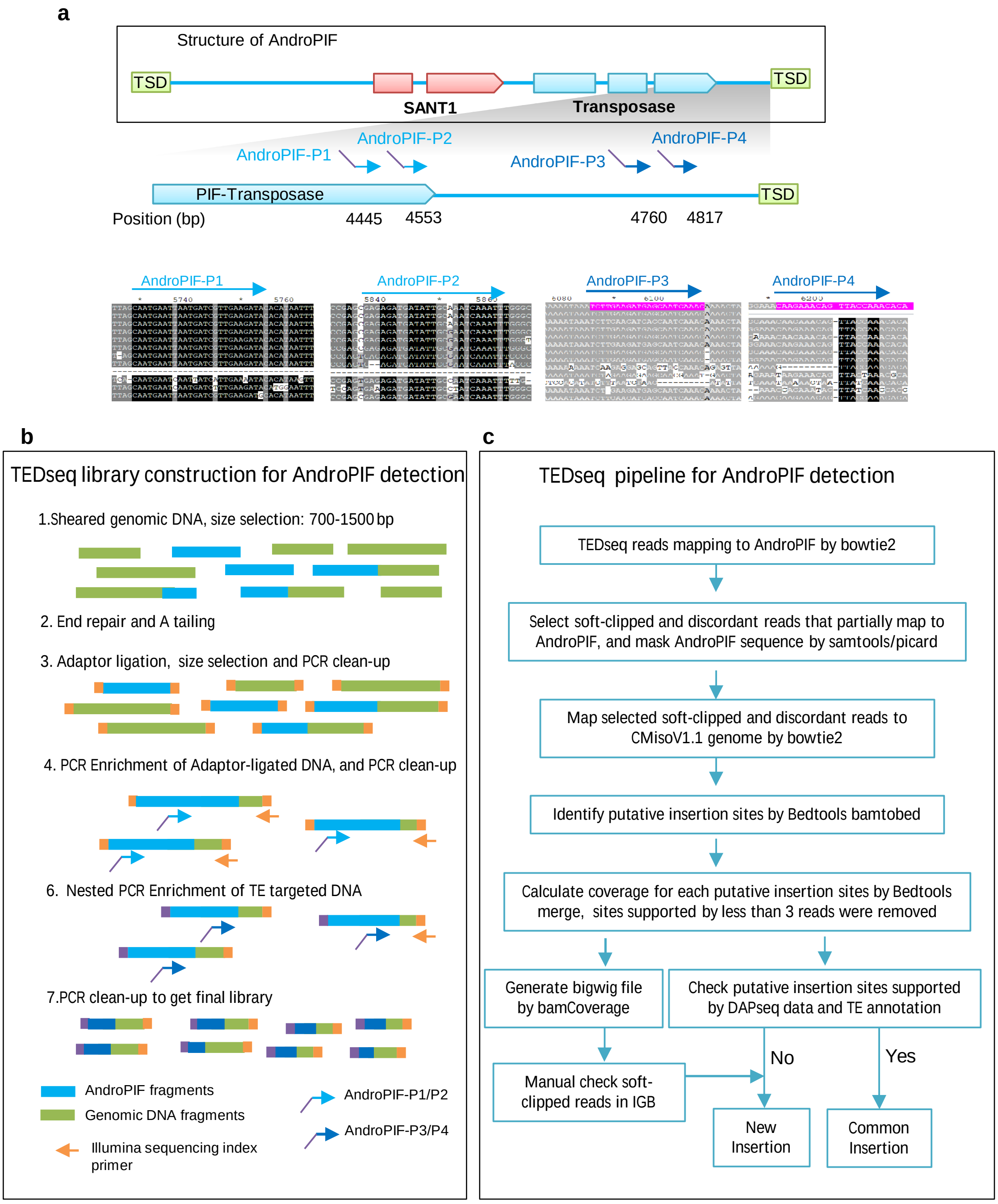

Figure S8. Process of TEDseq analysis of AndroPIF related elements

a Schematic diagram of primer design for AndroPIF capture TEDseq. b TEDseq library construction for AndroPIF detection in melon genomes. c TEDseq pipeline for AndroPIF detection.

a

replication	Percent of reads mapping to *androPIF* reference sequence	Percent of split-clipped and discordant-pair reads mapping to Mono genome	Number of detected PILs	Number of PILs identified in rep1 and rep2	Reads of *AndroPIF* inserted in *CmEIN2*
rep1	71.40%	95.58%	367	341	10
rep2	70.24%	97.14%	496		24

b

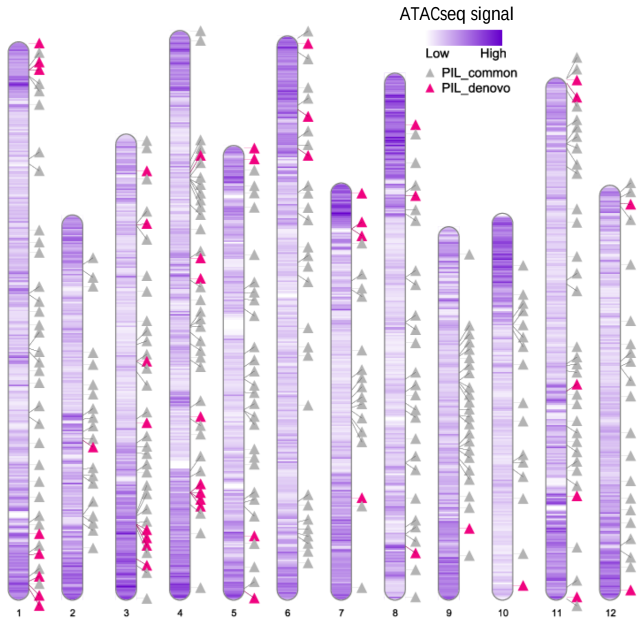

c

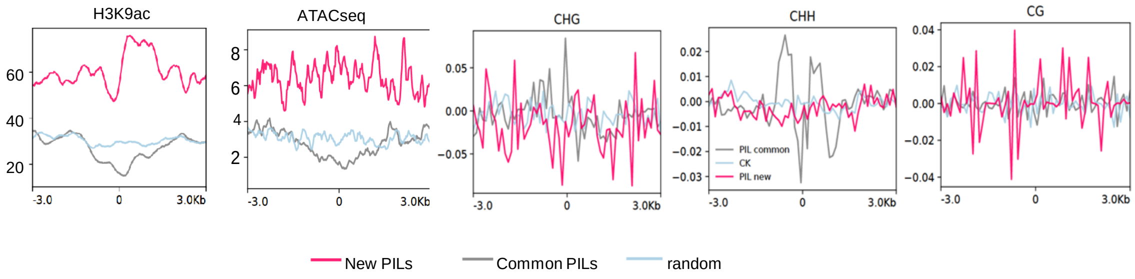

Figure S9. Data summary of TED-seq and associated chromatin features

a TED-seq data summary. AndroPIF insertion in EIN2 5'UTR was used as a positive control. Rep1 and rep2, are two independent assays using a pool of 50 CAM plants plus CAM106 as control. **b** Genomic positions of PILs and ATACseq signal density. **c** Level of H3K9ac, ATACseq, and DNA methylation in CHG, CHH, and CG context, in ±3kb sequences flancking insertion sites of Common PIFs, new PIFs, and random genomic regions (random).

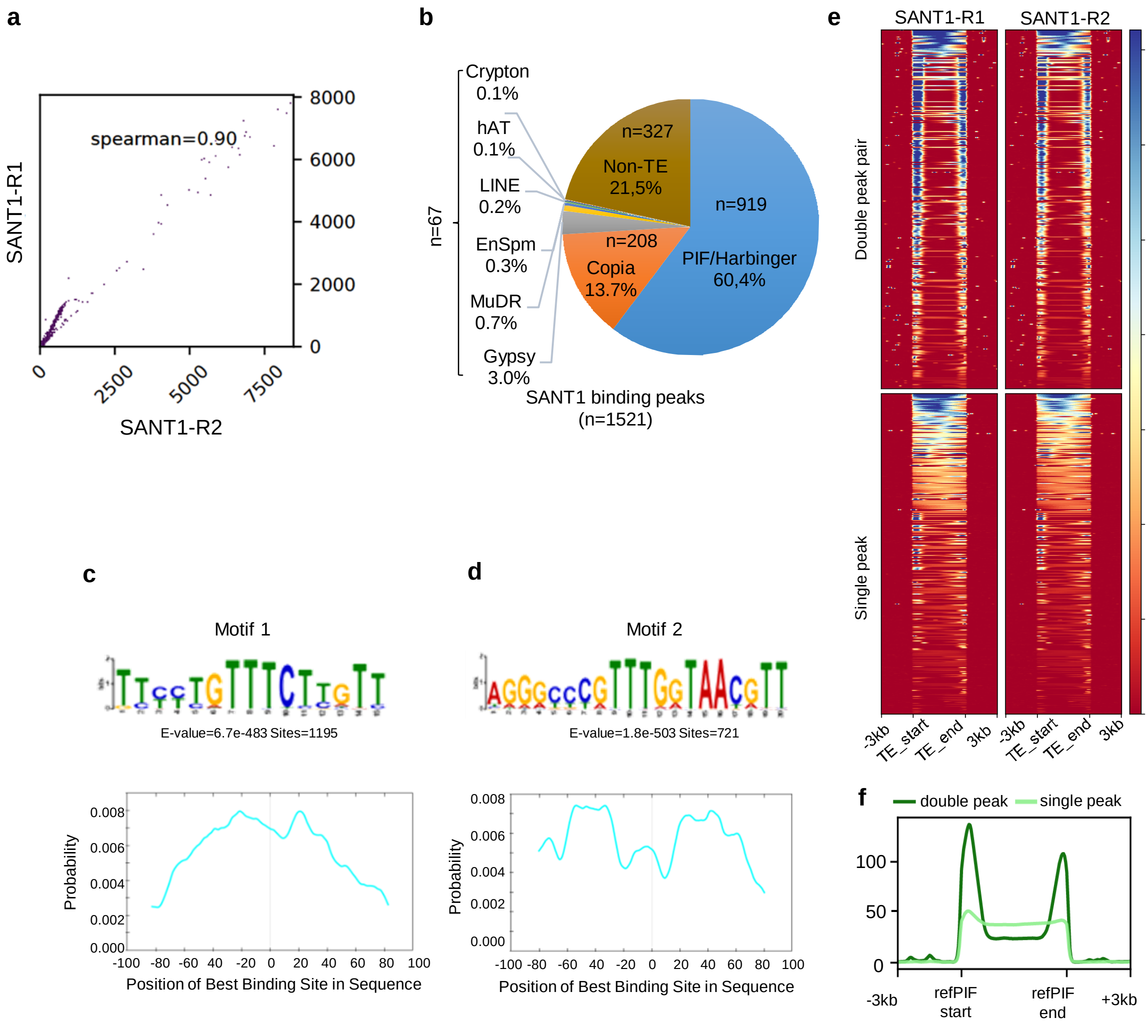

Figure S10. Data summary of SANT1 DAPseq analysis

a RPKM scatter plot, showing correlation between R1 and R2 SANT1 ampDAPseq assays. Spearman correlation coefficient is indicated on the plot. **b** Sequence annotation of SANT1-binding sites displayed in percent. **c,d** Top motifs enriched in SANT1 binding sites, and CentriMo plots highlighting the position of the motifs relative to the summit of SANT1 binding peaks. **e,f** Heatmap (e) and metaplot (f) showing SANT1 binding signals relative to Reference PIF loci

a

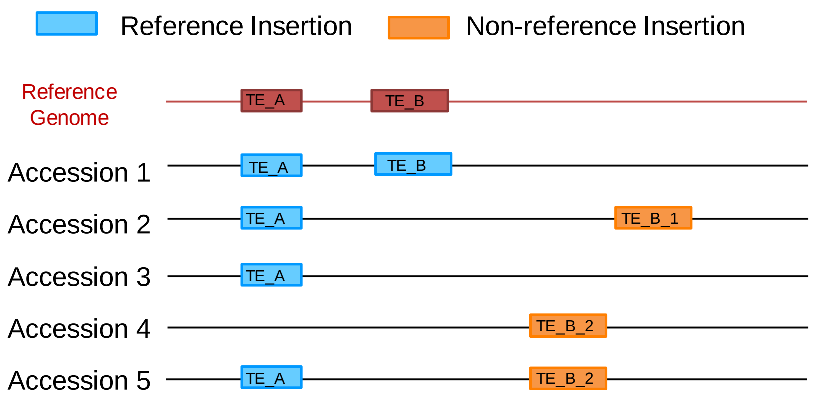

b

TIR sequences	SANT1 peak pairs (total=302)	Number of PIF element		
		2 borders supported by annotations	1 border supported by annotations	Not supported by annotations
TIR perfect much	54	54	0	0
TIR 1 mismatch	126	126	0	0
TIR 2 mismatch	184	182	1	1
TIR 3 mismatch	238	234	3	1
TIR 4mismatch	277	260	14	3
TIR Not found	25	17	4	4

c

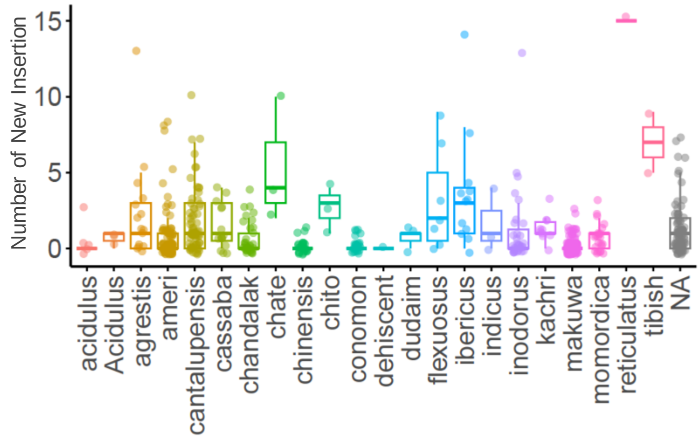

Figure S11. ***AndroPIF*** **elements polymorphism analysis based on reference and non-reference TE insertions**
a Schematic diagram of reference and non-reference *PIF/Harbinger* insertions. **b** Summary of the number of reference *androPIF* elements identified by *in silico* analysis, *or* manually annotated, by searching TIR sequences within SANT1 DAPseq peaks. The analysis allow 1 to 4 mismatches in 'GGGCCCGTTTG', androPIF reference TIR sequence. A total of 182 *androPIF* elements were identified, and classified as full length PIF. **c** Number of new insertion per accession in different melon botanical groups.

Supplementary Table S1: EMS-induced TILLING mutations in *CmEIN2*

Mutations in gDNA	Mutations in cDNA	Type of Mutations	AA changes	Phenotypes
G1175A	-	Splicing		Androecious
G1246A	-	Splicing		Androecious
C2518T	C1324T	Missense	L442F	Monoecious
C2570T	C1376T	Missense	T459I	Monoecious
G2854A	G1660A	Missense	E554K	Monoecious
G3118A	G1924A	Missense	G642R	Monoecious
C3155T	C1960T	Missense	L654F	Monoecious
G3159A	G1964A	Missense	S655T	Monoecious
G3490A	G2296A	Missense	D766N	Monoecious
G3601A	G2407A	Missense	V803I	Monoecious
G3643A	G2449A	Missense	D817N	Monoecious
G3713A	G2519A	Missense	G840D	Monoecious
C3865T	C2671T	Missense	L891F	Monoecious
C3971T	C2777T	Missense	S926F	Monoecious
G4417A	G3223A	Missense	E1075K	Monoecious
C4536T	C3341T	Missense	S1114F	Monoecious
C5282A	C3521T	Missense	P1174L	Monoecious
G5486A	C3725T	Missense	A1242V	Monoecious
G5515A	G3754A	Missense	E1252K	Monoecious

Supplementary Table S2: List of primers used in this study.

Name	Sequence	Purpose
Chr8_3820_F	GTCTAATTATTAATAGTAAACAATACCATG	Positional cloning
Chr8_3820_R	GTAAAATTGGTGAAATGGTGTCGTATG	Positional cloning
Chr8_1725_F	GCTTTACGAAAGAAAAGGATAGCAAGC	Positional cloning
Chr8_1725_R	GTGTATAAAACATCTTGGAAGGGTGAG	Positional cloning
Chr8_2871_F	GATCGTTTGTCAAAGTTACACGATCATTTAG	Positional cloning
Chr8_2871_R	GTCCCTTACTGGGTATATCTTATACTCAC	Positional cloning
Chr8_6571_F	GTGAGCACCTTCCCCGCTAGTAATCCGAGTG	Positional cloning
Chr8_6571_R	CAATTCTCCCATTTCCACTCTGCTTTC	Positional cloning
Chr8_4840_F	CATCTGACTTTAATGGACCAAGAAAGC	Positional cloning
Chr8_4840_R	CGACGATCCATTATAGTGGAGGATCCAT	Positional cloning
Chr8_7220_F	TGGTGTTTGTGTGCTAAATGGTTGCTTCTTAGT	Positional cloning
Chr8_7220_R	TGAAGCCTCCAAAGCAACTAGTGAATTAAGGT	Positional cloning
EIN2-qPCR-5UTR -F	AAGCCAATACGAAAACCCAGGTCAT	Quantitaitve RT-PCR
EIN2-qPCR-5UTR-R	AGAGACGATCAGGAATAGCTGTTTT	Quantitaitve RT-PCR
CmACS7 qPCR F2	CCTTACTATCCTGGATTTGACAGAGA	Quantitaitve RT-PCR
CmACS7 qPCR R2	TGGAGCGTTGGATCGTTGCTC	Quantitaitve RT-PCR
Cm_GENE-A_F	GAAAGAGGAGATTGATGTTGGAGT	Quantitaitve RT-PCR
Cm_GENE-A_R	TTCAAAAGATGCCTCATATCCAC	Quantitaitve RT-PCR
CmWIP1F	TAGGGCTTCCAACTCCTTCCTCTT	Quantitaitve RT-PCR
CmWIP1R	CTTGCAATTGATGGGTGTGATCTTCTTG	Quantitaitve RT-PCR
CmAct2_qPCR_F	ATTCTTGCATCTCTAAGTACCTTCC	Quantitaitve RT-PCR
CmAct2_qPCR_R	CCAACTAAAGGGAAATAACTCACC	Quantitaitve RT-PCR
SANT_exon12_2-F	GCAATGGAATGAGCTTTTTAAGGTG	Quantitaitve RT-PCR
SANT_exon2-R	CTTCAACACCAACGTTTGTGTTGCTT	Quantitaitve RT-PCR
Tpase_exon1-F	CTCTAGCTTTTAACAAGGTTTTGAGG	Quantitaitve RT-PCR
Tpase_exon12-R	CCATCAATAGCACCAATACAATCCTT	Quantitaitve RT-PCR
attb1_SANT1_F1	GGGGACAAGTTTGTACAAAAAAGCAGGCTTCA<br>TGTCACAAAAATCAACTGAACAAC	DAPseq
attb2R_ SANT1_R1	GGGGACCACTTTGTACAAGAAAGCTGGGTCCT<br>AAAGACTATTTATGAAATCTCTC	DAPseq
AndroPIF-P1	AATGATACGGCGACCACCGAGATCTACACTCT<br>TTCCCTACACGACGCTCTTCCGATCTCAATGAA<br>TTAATGATCGTTGAAGAT (Adaptor sequence is<br>underlined)	TEDseq
AndroPIF-P2	AATGATACGGCGACCACCGAGATCTACACTCT<br>TTCCCTACACGACGCTCTTCCGATCTGAGAGAT<br>GATATTRYVAATCAAATTT	TEDseq
AndroPIF-P3	AATGATACGGCGACCACCGAGATCTACACTCT<br>TTCCCTACACGACGCTCTTCCGATCTTCTTGAA<br>GATGAGCAATCAAACAA	TEDseq
AndroPIF-P4	AATGATACGGCGACCACCGAGATCTACACTCT<br>TTCCCTACACGACGCTCTTCCGATCTCAAGAAA<br>CAGTTACCAAACACATT	TEDseq
Illumina P7 sequencing index primer	5´-CAAGCAGAAGACGGCATACGAGAT**XXXXXX**<br>GTGACTGGAGTTCAGACGTGTGCTCTTCCGATC<br>-s-T-3´ (**XXXXXX** is specific sequencing index)	TEDseq
