# Supplementary material for: Harbinger transposon insertion in ethylene signaling gene leads to emergence of new sexual forms in cucurbits
Source: Nat Commun. 2024 Jun 7;15:4877. doi: 10.1038/s41467-024-49250-9 (PMC11161486; doi:10.1038/s41467-024-49250-9)
Supplement: Supplementary file 3 — Description of additional supplementary files [file 41467_2024_49250_MOESM3_ESM.pdf]

<u>Description of Additional Supplementary Files</u>

Supplementary Data 1: DEGs list identified by RNAseq

Supplementary Data 2: PILs identified by TEDseq

Supplementary Data 3: SANT1 binding sites identified by DAPseq

Supplementary Data 4: RefPIFs annotated in Mono genome

Supplementary Data 5: Annotated intact AndroPIFs

Supplementary Data 6: Summary of the sampled collection of melon accessions used for AndroPIF insertion analysis
