# Supplementary material for: Harbinger transposon insertion in ethylene signaling gene leads to emergence of new sexual forms in cucurbits
Source: Nat Commun. 2024 Jun 7;15:4877. doi: 10.1038/s41467-024-49250-9 (PMC11161486; doi:10.1038/s41467-024-49250-9)
Supplement: Supplementary file 10 — Reporting Summary [file 41467_2024_49250_MOESM10_ESM.pdf]

nature portfolio

Corresponding author(s): Abdelhafid Bendahmane

Last updated by author(s): Apr 22, 2024

Reporting Summary

Nature Portfolio wishes to improve the reproducibility of the work that we publish. This form provides structure for consistency and transparency in reporting. For further information on Nature Portfolio policies, see our Editorial Policies and the Editorial Policy Checklist.

Statistics

For all statistical analyses, confirm that the following items are present in the figure legend, table legend, main text, or Methods section.

n/a	Confirmed	
☐	☒	The exact sample size (*n*) for each experimental group/condition, given as a discrete number and unit of measurement
☐	☒	A statement on whether measurements were taken from distinct samples or whether the same sample was measured repeatedly
☐	☒	The statistical test(s) used AND whether they are one- or two-sided<br>*Only common tests should be described solely by name; describe more complex techniques in the Methods section.*
☒	☐	A description of all covariates tested
☒	☐	A description of any assumptions or corrections, such as tests of normality and adjustment for multiple comparisons
☐	☒	A full description of the statistical parameters including central tendency (e.g. means) or other basic estimates (e.g. regression coefficient) AND variation (e.g. standard deviation) or associated estimates of uncertainty (e.g. confidence intervals)
☐	☒	For null hypothesis testing, the test statistic (e.g. *F*, *t*, *r*) with confidence intervals, effect sizes, degrees of freedom and *P* value noted<br>*Give P values as exact values whenever suitable.*
☒	☐	For Bayesian analysis, information on the choice of priors and Markov chain Monte Carlo settings
☒	☐	For hierarchical and complex designs, identification of the appropriate level for tests and full reporting of outcomes
☒	☐	Estimates of effect sizes (e.g. Cohen's *d*, Pearson's *r*), indicating how they were calculated

Our web collection on statistics for biologists contains articles on many of the points above.

Software and code

Policy information about availability of computer code

Data collection

1. RT-qPCR data were collected by using CFX96 Real-time PCR system(Bio-Rad).
2. RNA-seq data were collected by using Illumina NovaSeq 6000 system.
3. Bulk genomic DNA sequencing data were collected by using Illumina HiSeq2000 system
4. TEDseq libraries were sequenced using Illumina Miseq system
5. BiSulfite-seq libraries were sequenced using Illumina NovaSeq 6000 system
6. AmpDAP-seq libraries were sequenced using Illumina NextSeq500 system

Data analysis

1. RNA-Seq data quality was assessed using FastQC (v0.11) and STAR (v2.7.5c)has been used to generate the mapping files. The mapped reads were assigned to genes with featureCount (v2.0.3). DiCoExpress was used to identify the differentially expressed genes, DEGs (adjusted p-value <0.05).
2. GO enrichments were completed by ClusterProfiler(v4.6.2) with customized GO annotation.
3. Bulk genomic DNA sequences were trimmed by using Trimmomatic (v0.39) and paired-reads were mapped to the melon reference genome using CLC-Genomics workbench 12.2.2 software.
4. BS-seq data were analysed by MethylStar(v1.4) pipeline. The differentially methylated sites were identified by methylkit and DMRcaller.
5. AmpDAP-seq data were trimmed using Trimmomatic (version 0.39) and then mapped to the melon genome using Bowtie2v2.2.6. Duplicated reads were marked and removed using SAMtools. Aligned reads were analyzed for peak calling using MACS2 (v. 2.2.7.1).
6. Transposable elements insertion detection was performed using the TEFLoN method and the McClintock(v2.0.0) pipeline.
7. TEDseq data were mapped to AndroPIF and then to the melon genome using Bowtie2(v2.3.5). TE target site analysis was performed by R(v4.2.2). The level of epigenetic marks around the TE insertion sites were calculated by Deeptools(v3.5.5).
8. Two-tailed unpaired Student's t-test and one-way or two-way ANOVA (with Tukey's post hoc test) were calculated using R(v4.2.2) or

Graphpad Prism(v9.3.1).
9. Bar graphs overlaid with dot plots were generated by using Graphpad Prism (v9.3.1)
10. Gene primers was designed using the Primer3 software v.4.1.0 (https://primer3.ut.ee)

For manuscripts utilizing custom algorithms or software that are central to the research but not yet described in published literature, software must be made available to editors and reviewers. We strongly encourage code deposition in a community repository (e.g. GitHub). See the Nature Portfolio guidelines for submitting code & software for further information.

Data

Policy information about availability of data

All manuscripts must include a data availability statement. This statement should provide the following information, where applicable:

- Accession codes, unique identifiers, or web links for publicly available datasets
- A description of any restrictions on data availability
- For clinical datasets or third party data, please ensure that the statement adheres to our policy

Sequencing data generated in this study have been deposited in the NCBI Sequence Read Archive database under the accession number PRJNA1085522[https://www.ncbi.nlm.nih.gov/bioproject/PRJNA1085522], including RNA-Seq, Bisulfite-Seq, TEDseq, and AmpDAP-seq. Previously published data used in this study are SRX15631305[https://www.ncbi.nlm.nih.gov/sra/?term=SRX15631305], SRX15631306[https://www.ncbi.nlm.nih.gov/sra/?term=SRX15631306], and PRJNA383830[https://www.ncbi.nlm.nih.gov/bioproject/PRJNA383830/]. Source data are provided with this paper.

Research involving human participants, their data, or biological material

Policy information about studies with human participants or human data. See also policy information about sex, gender (identity/presentation), and sexual orientation and race, ethnicity and racism.

Reporting on sex and gender	N.A.
Reporting on race, ethnicity, or other socially relevant groupings	N.A.
Population characteristics	N.A.
Recruitment	N.A.
Ethics oversight	N.A.

Note that full information on the approval of the study protocol must also be provided in the manuscript.

Field-specific reporting

Please select the one below that is the best fit for your research. If you are not sure, read the appropriate sections before making your selection.

☒ Life sciences ☐ Behavioural & social sciences ☐ Ecological, evolutionary & environmental sciences

For a reference copy of the document with all sections, see nature.com/documents/nr-reporting-summary-flat.pdf

Life sciences study design

All studies must disclose on these points even when the disclosure is negative.

Sample size	Sample size is mentioned in the respective figure legends. The sample size was determined based on previous publications in similar scientific studies, based on the feasibility of sample collection and to ensure significance and reproducibility to the statistical tests. For RNA-seq, ampDAP-seq, Whole Genome Bisulfite sequencing, 2 or 3 biological replicates were collected. The qPCR analysis had sample sizes of above 3 independent experiments. The plant phenotyping had sample sizes of above 5 independent experiments.
Data exclusions	No data was excluded
Replication	For each experiment, the number of biological replicates is indicated in the respective figure legends. All experiments were biologicallyrepeated at least three times, with similar results.
Randomization	The different melon and cucumber genotypes were randomly distributed and grown in different greenhouses in different geographic region,Paris area and Avignon area.
Blinding	Not applicable since no group allocation was conducted in this study.

Reporting for specific materials, systems and methods

We require information from authors about some types of materials, experimental systems and methods used in many studies. Here, indicate whether each material, system or method listed is relevant to your study. If you are not sure if a list item applies to your research, read the appropriate section before selecting a response.

Materials & experimental systems

n/a	Involved in the study
☒	☐ Antibodies
☒	☐ Eukaryotic cell lines
☒	☐ Palaeontology and archaeology
☒	☐ Animals and other organisms
☒	☐ Clinical data
☒	☐ Dual use research of concern
☐	☒ Plants

Methods

n/a	Involved in the study
☒	☐ ChIP-seq
☒	☐ Flow cytometry
☒	☐ MRI-based neuroimaging

Plants

Seed stocks	INRAE Centre for Vegetable Germplasm, GAFL, Avginon, France
Novel plant genotypes	Melon variety 'CAM' WT and ein2 mutant. CharMono EMS collections.
Authentication	CharMono EMS collections are generated by EMS treatment.
